# Acylated and desacyl ghrelin are associated with hepatic lipogenesis, β-oxidation and autophagy: role in NAFLD amelioration after sleeve gastrectomy in obese rats

**DOI:** 10.1038/srep39942

**Published:** 2016-12-23

**Authors:** Silvia Ezquerro, Leire Méndez-Giménez, Sara Becerril, Rafael Moncada, Víctor Valentí, Victoria Catalán, Javier Gómez-Ambrosi, Gema Frühbeck, Amaia Rodríguez

**Affiliations:** 1 Metabolic Research Laboratory, Clínica Universidad de Navarra, Pamplona, Spain; 2 CIBER Fisiopatología de la Obesidad y Nutrición (CIBEROBN), Instituto de Salud Carlos III, Madrid, Spain; 3 Obesity & Adipobiology Group, Instituto de Investigación Sanitaria de Navarra (IdiSNA), Pamplona, Spain; 4 Department of Anesthesia, Clínica Universidad de Navarra, Pamplona, Spain; 5 Department of Surgery, Clínica Universidad de Navarra, Pamplona, Spain; 6 Department of Endocrinology & Nutrition, Clínica Universidad de Navarra, Pamplona, Spain

## Abstract

Bariatric surgery improves non-alcoholic fatty liver disease (NAFLD). Our aim was to investigate the potential role of ghrelin isoforms in the resolution of hepatic steatosis after sleeve gastrectomy, a restrictive bariatric surgery procedure, in diet-induced obese rats. Male Wistar rats (n = 161) were subjected to surgical (sham operation and sleeve gastrectomy) or dietary interventions [fed *ad libitum* a normal (ND) or a high-fat (HFD) diet or pair-fed]. Obese rats developed hepatosteatosis and showed decreased circulating desacyl ghrelin without changes in acylated ghrelin. Sleeve gastrectomy induced a dramatic decrease of desacyl ghrelin, but increased the acylated/desacyl ghrelin ratio. Moreover, sleeve gastrectomy reduced hepatic triglyceride content and lipogenic enzymes *Mogat2* and *Dgat1*, increased mitochondrial DNA amount and induced AMPK-activated mitochondrial FFA β-oxidation and autophagy to a higher extent than caloric restriction. In primary rat hepatocytes, the incubation with both acylated and desacyl ghrelin (10, 100 and 1,000 pmol/L) significantly increased TG content, triggered AMPK-activated mitochondrial FFA β-oxidation and autophagy. Our data suggest that the decrease in the most abundant isoform, desacyl ghrelin, after sleeve gastrectomy contributes to the reduction of lipogenesis, whereas the increased relative acylated ghrelin levels activate factors involved in mitochondrial FFA β-oxidation and autophagy in obese rats, thereby ameliorating NAFLD.

Non-alcoholic fatty liver disease (NAFLD) is a pathology characterized by intrahepatic triacylglycerol (TG) overaccumulation^1^,^2^, which is commonly associated with obesity, dyslipidemia, insulin resistance and type 2 diabetes^3^. NAFLD comprises a spectrum of liver disorders ranging from steatosis to non-alcoholic steatohepatitis (NASH) with risk of progression to liver cirrhosis and hepatocellular carcinoma^4^. The prevalence of NAFLD and NASH increases from around 20% and 3%, respectively, in the general population to 75% and 25–70%, respectively, in morbid obesity^5^,^6^. The excessive TG deposition in hepatocytes derives from an increased delivery of free fatty acids (FFA) into the liver from several different sources: excess dietary fat, higher FFA release from adipocytes via lipolysis, as well as increased *de novo* hepatic lipogenesis, ultimately leading to the development of obesity-associated NAFLD^7^. In addition, growing evidence indicates that hepatic mitochondrial dysfunction as well as alterations in autophagy are also involved in the development of this disease^8^. Bariatric surgery constitutes an effective treatment for morbid obesity achieving a more sustainable weight loss than that observed with lifestyle changes or pharmacological therapy^9^. Moreover, this procedure significantly improves, or even reverses, NAFLD, NASH and fibrosis in obese patients^10^,^11^,^12^. However, the molecular mechanisms underlying surgically-induced improvement of hepatic function remain unclear.

The orexigenic hormone ghrelin has been linked with the development of hepatosteatosis and the progression to NASH^13^. Ghrelin is a 28 amino acid peptide mainly synthesized in X/A cells of the oxyntic glands in the mucosa layer of the gastric fundus and circulates in two main forms: acylated ghrelin, with an *n*-octanoyl group at the serine 3 residue, and desacyl ghrelin, without this post-translational modification^14^,^15^,^16^. Administration of exogenous ghrelin increases food intake by activating hypothalamic neuropeptide Y/agouti-related peptide-containing neurons, which also express GHS-R type 1a, via the modulation of fatty acid metabolism^17^,^18^. Ghrelin also stimulates adiposity peripherally, through a direct stimulation of adipogenesis and lipogenesis in murine and human adipocytes^19^,^20^. The liver also constitutes a target for the lipogenic actions of ghrelin, since both chronic central and peripheral administration of ghrelin stimulate hepatic lipid storage^21^,^22^. In addition, opposite effects of ghrelin isoforms on hepatic glucose metabolism have been reported with desacyl ghrelin suppressing glucose release by hepatocytes as well as antagonizing the acylated ghrelin-induced increase in hepatic glucose output *in vitro*^23^. NAFLD is associated with alterations in total ghrelin concentrations^24^,^25^, but the specific role of acylated and desacyl in hepatic lipid metabolism has not been disentangled.

Circulating ghrelin concentrations decrease after sleeve gastrectomy due to the resection of the gastric fundus^15^ with RYGB additionally suppressing post-prandial ghrelin levels^26^. The aim of the present study was to analyze in diet-induced obese rats the implication of ghrelin isoforms in the improvement of hepatosteatosis after sleeve gastrectomy, a restrictive bariatric surgery procedure. In this regard, the potential differences in circulating acylated and desacyl ghrelin concentrations in obesity and after sleeve gastrectomy as well as their association with hepatic lipogenesis, lipophagy and mitochondrial FFA β-oxidation were evaluated. Moreover, the direct effects of the major isoforms of ghrelin on key regulatory molecules involved in these pathways were also studied in primary rat hepatocytes.

## Results

### Sleeve gastrectomy reduced serum desacyl ghrelin, but not the acylated form

**Figure 1:**
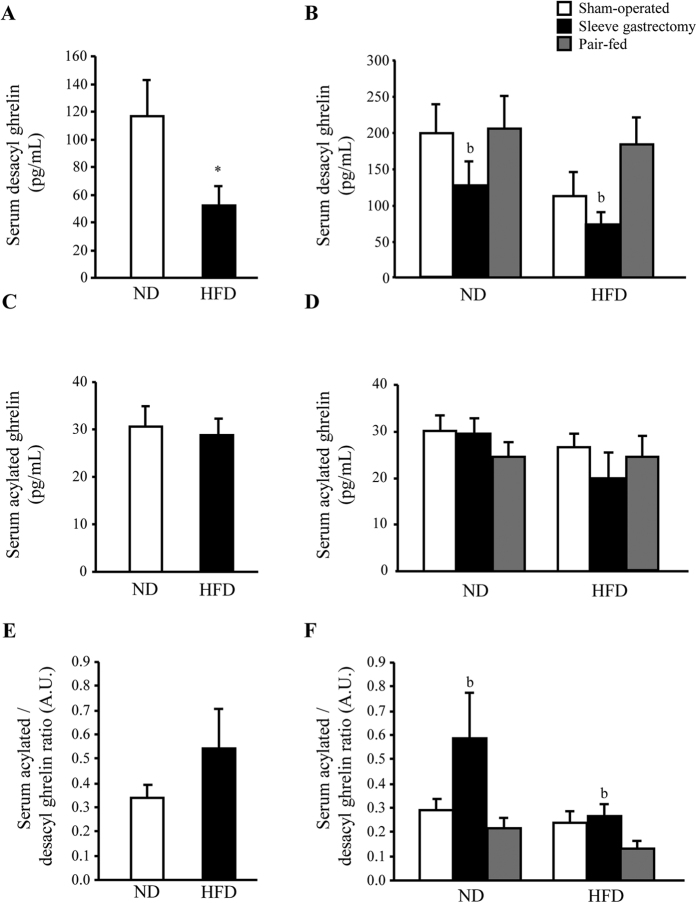
Effect of sleeve gastrectomy on circulating ghrelin isoforms. Bar graphs illustrate the impact of obesity and sleeve gastrectomy-induced weight loss on serum desacyl (**A**,**B**) and acylated (**C**,**D**) ghrelin levels as well as the acylated/desacyl ghrelin ratio (**E**,**F**). Differences were analyzed by Student’s *t* test or two-way ANOVA, where appropriate. *P < 0.05 *vs* lean control ND; ^b^*P* < 0.05 effect of surgery.

Sleeve gastrectomy improved body weight, adiposity and insulin sensitivity of experimental animals (Tables 1 and 2). As expected, serum desacyl ghrelin was decreased in obese rats (*P* < 0.05) (Fig. 1A), but no significant changes in circulating acylated ghrelin and the acylated/desacyl ghrelin ratio were found between lean and obese rats (Fig. 1C and E). Rats undergoing bariatric surgery exhibited decreased (*P* < 0.05) desacyl ghrelin levels, without changes in acylated ghrelin concentrations (*P* = 0.820) (Fig. 1B and D). An increase in the acylated/desacyl ghrelin ratio (*P* < 0.05) was found after surgery (Fig. 1F).

### Sleeve gastrectomy improved hepatic function and steatosis through the regulation of lipogenic factors

**Figure 2:**
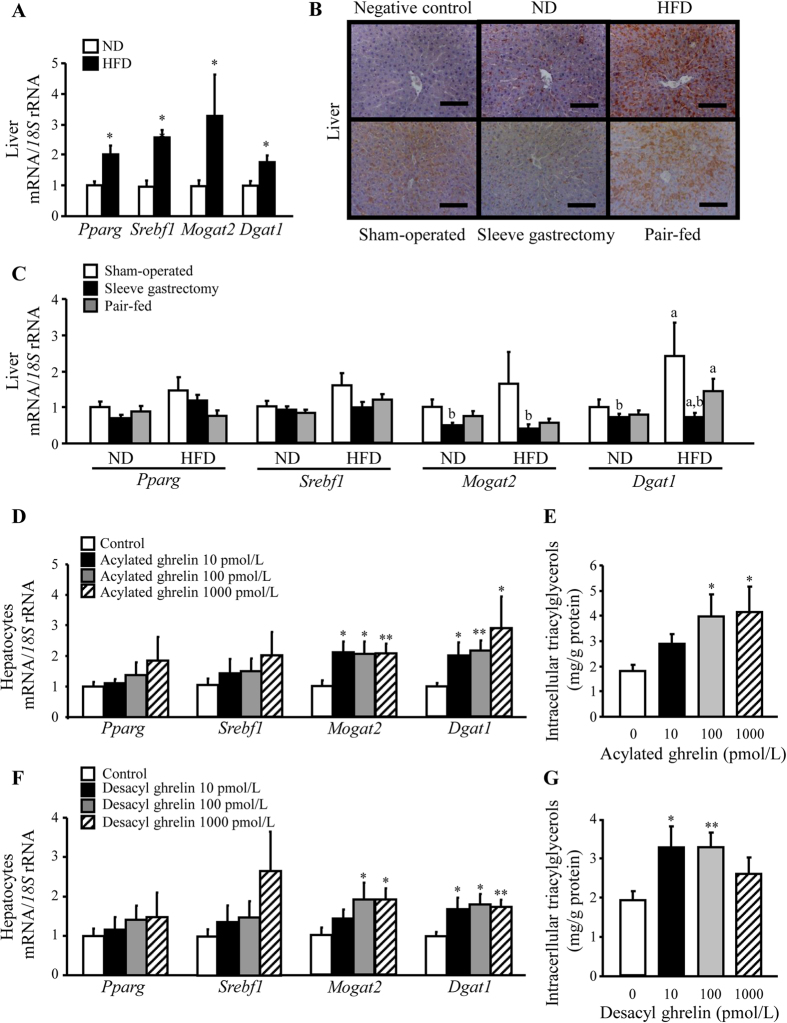
Effect of acylated and desacyl ghrelin on the improvement of hepatic steatosis after sleeve gastrectomy. Impact of obesity (**A**) and sleeve gastrectomy (**C**) on the mRNA expression levels of *Pparg, Srebf1, Mogat2* and *Dgat1* in liver samples of experimental animals. (**B**) Immunohistochemical detection of adipophilin in histological sections of rat liver (magnification 200x, *scale bar* = 100 μm). Effect of acylated (**D**,**E**) and desacyl (**F**,**G**) ghrelin on key lipogenic factors and intracellular triglycerides in rat hepatocyte cultures. The gene expression in lean rats, in the sham-operated groups fed a ND and in unstimulated hepatocytes was assumed to be 1. *P < 0.05; **P < 0.01 *vs* lean control ND or unstimulated hepatocytes; ^a^*P* < 0.05 effect of diet; ^b^*P* < 0.05 effect of surgery.

Obesity was associated with higher liver weight (*P* < 0.05), intrahepatic TG (*P* < 0.001) and serum AST and ALT levels, although the differences in transaminases did not read statistical significance (Table 1). Moreover, obese animals showed higher (*P* < 0.05) transcript levels of the lipogenic genes *Pparg, Srebf1, Mogat2* and *Dgat1* as well as a moderate-to-severe hepatic steatosis evidenced by the staining of the lipid droplet-coating protein adipophilin in liver sections (Fig. 2A–B and Supplemental Fig. 1). Liver weight (*P* < 0.01), intrahepatic TG (*P* < 0.05) and AST levels (*P* < 0.001) were decreased in rats subjected to bariatric surgery (Table 2). Accordingly, sleeve-gastrectomized rats exhibited a tendency towards a downregulation in hepatic *Pparg (P* = 0.084) and *Srebf1 (P* = 0.153) transcription factors as well as lower (*P* < 0.05) mRNA levels of *Mogat2* and *Dgat1* and mild steatosis evidenced by adipophilin staining (Fig. 2B–C and Supplemental Fig. 1).

### Acylated and desacyl ghrelin stimulate lipogenesis in primary rat hepatocytes

We next examined whether acylated and desacyl ghrelin levels are associated with the improvement in hepatic steatosis after sleeve gastrectomy. A positive correlation between desacyl ghrelin and ALT (*r* = 0.23, *P* < 0.05) was found, whereas acylated ghrelin was negatively correlated with intrahepatic TG (*r* = *−*0.36, *P* < 0.001). The direct effect of both ghrelin isoforms on lipogenesis was also evaluated in primary rat hepatocytes. Both acylated and desacyl ghrelin increased (*P* < 0.05) the mRNA expression of *Mogat2* and *Dgat1* and intracellular TG content in rat hepatocytes (Fig. 2D–F), although no significant changes were observed in *Pparg* and *Srebf1* transcript levels.

### Acylated and desacyl ghrelin induced changes in factors related to AMPK-induced hepatic mitochondrial β-oxidation

**Figure 3:**
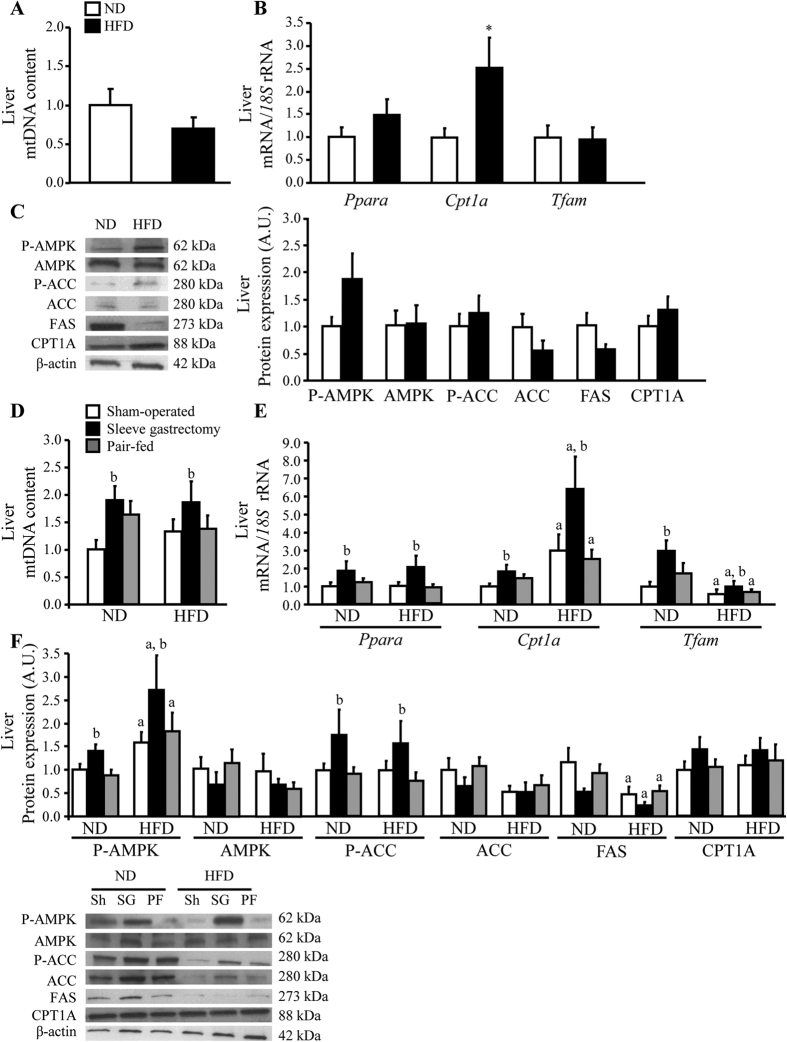
Impact of sleeve gastrectomy on hepatic mitochondrial biogenesis and FFA β-oxidation. Bar graphs show the effect of obesity (**A**,**B**) and sleeve gastrectomy (**D**,**E**) on the hepatic gene expression of molecules involved in mitochondrial biogenesis (mtDNA content and *Tfam*) and FFA β-oxidation (*Ppara* and *Cpt1a*). Representative cropped blots and Western-blot analysis show the impact of obesity (**C**) and sleeve gastrectomy (**F**) on the phosphorylation/activation of AMPK in Thr172 and the phosphorylation/inactivation of ACC in Ser79 as well as the basal expression of AMPK, ACC, FAS and CPT1A enzymes in liver samples of the experimental groups. The gene and protein expression in lean or in the sham group fed a ND was assumed to be 1. *P < 0.05 *vs* lean control ND; ^a^*P* < 0.05 effect of diet; ^b^*P* < 0.05 effect of surgery.

Since NAFLD is highly correlated with reduced mitochondrial lipid oxidation^27^, the effect of sleeve gastrectomy on the regulation of mitochondrial FFA β-oxidation pathway was evaluated. Obesity did not change the hepatic mitochondrial number, as evidenced by a similar mtDNA copy number and transcript levels of *Tfam*, a factor involved in mitochondrial genome replication (Fig. 3A–B). Interestingly, a significant (*P* < 0.05) increase in both markers of mitochondrial biogenesis was observed following sleeve gastrectomy (Fig. 3D–E). It is well established that activated AMPK directly phosphorylates and inactivates ACC, an enzyme that catalyzes the rate-limiting reaction for FFA synthesis^28^. The inhibition of ACC reduces the production of malonyl-CoA, an allosteric inhibitor of CPT1a, which is a key regulator of mitochondrial FFA uptake and its expression is positively regulated by PPARα^29^. Obese rats exhibited similar basal AMPK levels and AMPK phosphorylation/activation compared to lean rats (Fig. 3C). Accordingly, no differences (*P* > 0.05) in basal ACC levels, ACC phosphorylation/inactivation state as well as *Ppara,* CPT1A and FAS were observed in the obese group compared with lean rats (Fig. 3B–C). Interestingly, animals undergoing sleeve gastrectomy showed higher (*P* < 0.05) P-AMPK/AMPK and P-ACC/ACC ratios compared to the other interventional groups, as well as a marked upregulation (*P* < 0.05) of *Ppara* and *Cpt1a* expression, whereas CPT1A tended to increase (Fig. 3E–F).

**Figure 4:**
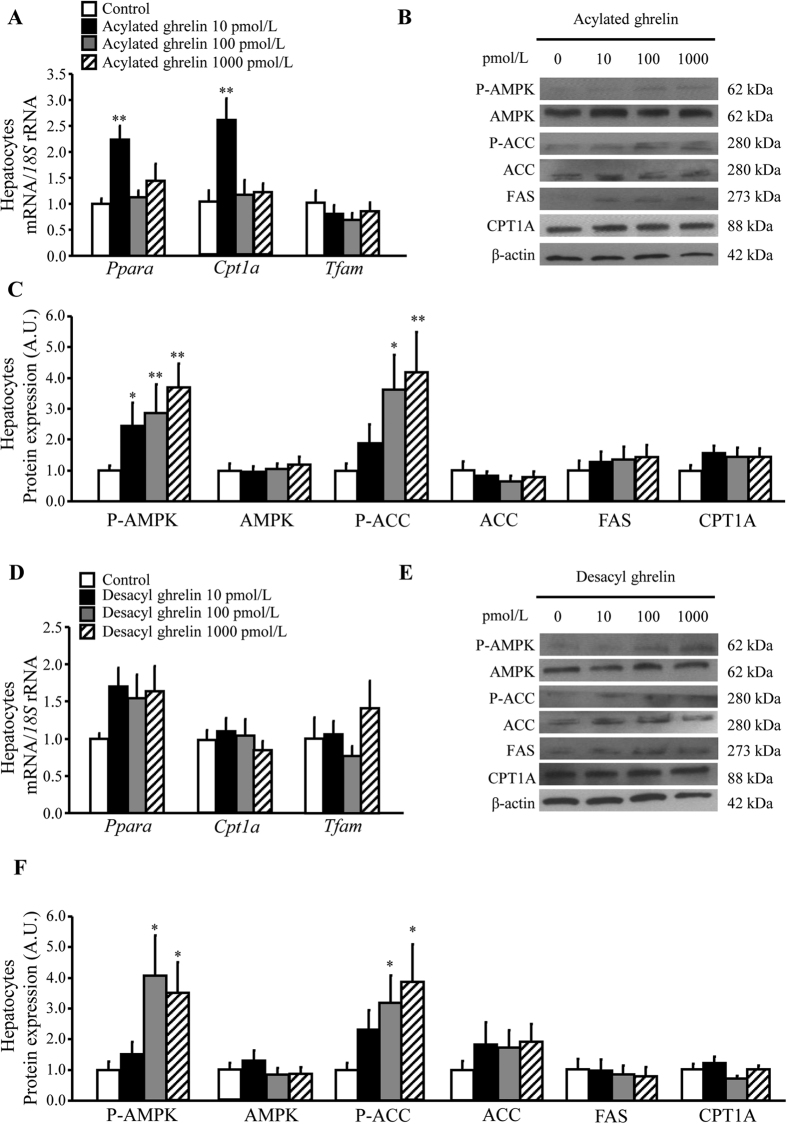
Effect of acylated and desacyl ghrelin on mitochondrial biogenesis and FFA β-oxidation. Bar graphs show the effect of acylated (**A**) and desacyl (**D**) ghrelin on the expression of molecules involved in mitochondrial biogenesis (*Tfam*) and FFA β-oxidation (*Ppara* and *Cpt1a*). Representative cropped blots and Western-blot analysis showing impact of acylated (**B**,**C**) and desacyl (**E**,**F**) ghrelin on P-AMPK/AMPK and P-ACC/ACC ratios as well as the basal expression of AMPK, ACC, FAS and CPT1A enzymes in primary cultures of rat hepatocytes. The gene and protein expression in unstimulated hepatocytes was assumed to be 1. *P < 0.05; **P < 0.01 *vs* unstimulated hepatocytes.

The ability of acylated and desacyl ghrelin to modulate mitochondrial FFA β-oxidation was also tested in rat hepatocytes. Both isoforms enhanced (*P* < 0.05) AMPK and ACC phosphorylation rates (Fig. 4C and F), without changing FAS and *Tfam* expression (Fig. 4A–F). Furthermore, the physiological concentration of acylated ghrelin significantly increased (*P* < 0.05) *Ppara* and *Cpt1a* transcripts (Fig. 4A), whereas the protein expression of CPT1A showed a similar trend only after acylated ghrelin stimulation, but differences did not reach statistical significance (Fig. 4C).

### Acylated ghrelin, and to a lesser extent desacyl ghrelin, activated hepatic autophagy

**Figure 5:**
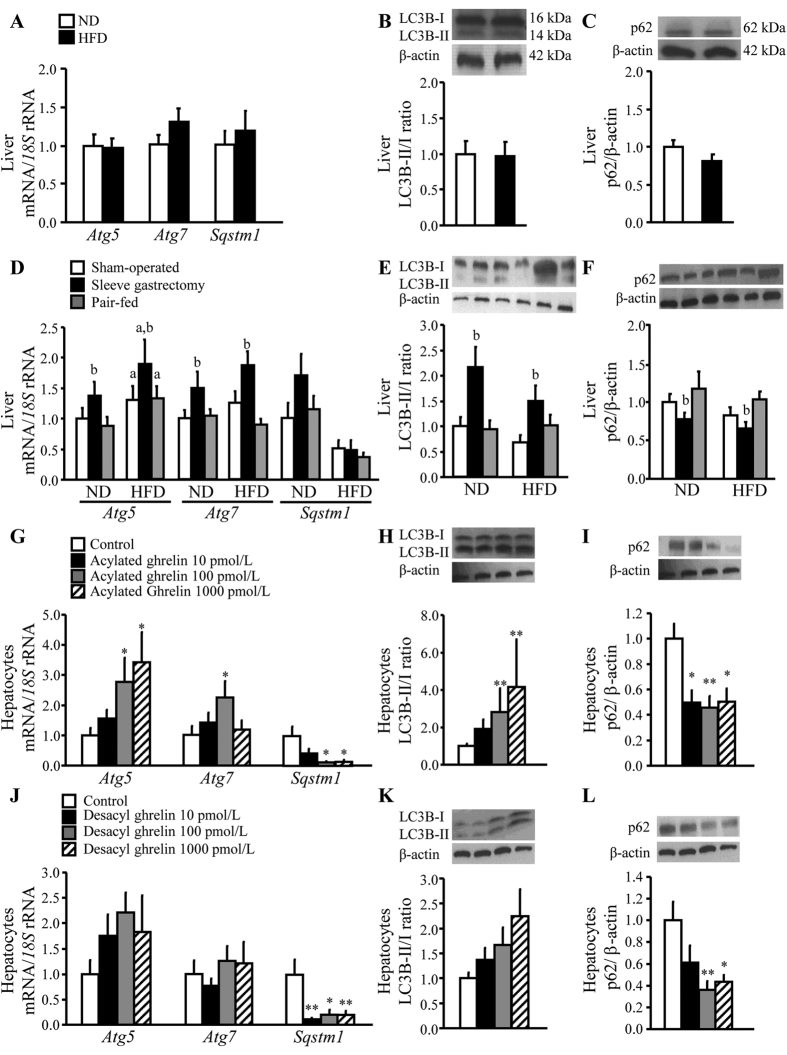
Impact of acylated and desacyl ghrelin on the activation of autophagy after sleeve gastrectomy. Effect of obesity and sleeve gastrectomy on the expression of *Atg5, Atg7* and *Sqstm1* genes (**A**,**D**), autophagosome formation as evidenced by the LC3B-II/I ratio (**B**,**E**) as well as autophagy inhibition determined by p62 accumulation (**C**,**F**) in liver samples of the experimental groups. Representative cropped blots are shown at the top of the figures. Effect of acylated (**G**,**H**,**I**) and desacyl (**J**,**K**,**L**) ghrelin on key autophagy factors in rat hepatocyte cultures. The gene expression in lean rats, in the sham-operated groups fed a normal diet and in unstimulated hepatocytes was assumed to be 1. ^a^*P* < 0.05 effect of diet; ^b^*P* < 0.05 effect of surgery. *P < 0.05; **P < 0.01 *vs* unstimulated hepatocytes.

The role of autophagy, featured by an increased LC3B II/I ratio associated to a decreased p62/SQSTM1 protein content^30^, on the improvement of NAFLD after sleeve gastrectomy was next analysed. Obese rats showed similar hepatic expression of the autophagy-related genes *Atg5* and *Atg7* as well as in the LC3B-II/I ratio and the p62 protein level than lean rats (Fig. 5A–C). By contrast, sleeve gastrectomy was associated with an increase in *Atg5* and *Atg7* mRNA (Fig. 5D) and LC3B-II/I ratio together with a down-regulation of p62 (*P* < 0.05) (Fig. 5E–F). Interestingly, the acylated/desacyl ghrelin ratio was positively correlated with hepatic transcript levels of *Atg5 (r* = 0.22, *P* < 0.05). Thus, we next determined whether acylated and desacyl ghrelin directly affect autophagy in rat hepatocytes. The stimulation of hepatic cells with acylated ghrelin upregulated (*P* < 0.05) the expression of ATGs and LC3B-II/I ratio (Fig. 5G–H), while p62 levels were markedly diminished (Fig. 5I). However, desacyl ghrelin only modified the expression of p62 (*P* < 0.01) (Fig. 5L).

## Discussion

Obesity is associated with an increased risk of NAFLD^1^,^2^ and surgically-induced weight loss improves serum transaminases and hepatic function^31^,^32^,^33^,^34^. In line with these observations, our data provide evidence that sleeve gastrectomy ameliorates hepatic function, as evidenced by an improved profile of AST and ALT, and hepatosteatosis, through the downregulation of lipogenic factors *Pparg, Srebf1, Mogat2* and *Dgat1.* However, the molecular mechanisms underlying this amelioration remain undefined. Ghrelin has been recently proposed as a potential link between obesity and NAFLD^35^. In spite of its orexigenic and adipogenic properties, circulating total ghrelin concentrations are paradoxically decreased in obesity^36^. Our findings show that obese rats exhibited lower serum desacyl ghrelin levels without changes in acylated ghrelin and acylated/desacyl ghrelin ratio, which is in agreement with previous reports^20^,^37^,^38^,^39^. Interestingly, obese individuals with low ghrelin levels are more prone to weight regain after following a hypocaloric diet intervention^40^, suggesting an impaired central and/or peripheral ghrelin signalling that might explain the high-fat feeding in these subjects. One limitation of our study is that we only measured fasting ghrelin concentrations, and changes in post-feeding ghrelin levels are also important to understand their overall role in the regulation of appetite and metabolic flexibility. As expected, sleeve gastrectomy reduced desacyl ghrelin levels, due to the resection of the gastric fundus, the major production site of the hormone^15^. By contrast, plasma acylated ghrelin remained unchanged after bariatric surgery, which is in accordance with other authors^26^,^41^, and the acylated/desacyl ghrelin ratio increased, suggesting an enhanced post-transcriptional modification in order to maintain the circulating levels of the acylated hormone. In a previous work of our group, we found that both acylated and desacyl ghrelin stimulated intracellular lipid accumulation in human differentiated omental adipocytes through the upregulation of PPARγ and SREBP-1c and other fat-storage related molecules^20^. Accordingly, we herein show that both ghrelin isoforms also increase intracellular TG content in rat hepatocytes by increasing the transcript levels of the lipogenic factors *Mogat2* and *Dgat1*. Since desacyl ghrelin represents ~90% of total ghrelin^20^,^42^, the amelioration of the hepatic function after sleeve gastrectomy might be partially due to the decrease of the desacylated hormone after the removal of the gastric fundus.

Sleeve gastrectomy was associated with an improvement in insulin sensitivity, as evidenced by lower insulinemia and HOMA, as well as lower glycemia, which is in accordance with previous results^34^. Opposite effects of acylated and desacyl ghrelin on insulin sensitivity have been reported. Although this might seem paradoxical, tight homeostatic control in some physiological processes is achieved by fine-tuning hormonal actions with opposite effects of the diverse isoforms^43^. Thus, exogenous desacyl ghrelin administration acts as a potent insulin secretagogue, whereas acylated ghrelin decreases glucose-induced insulin release and increases glucose levels in both humans and rodents^44^,^45^. In line with these observations, genetic, immunological, and pharmacological blockade of ghrelin signalling enhances glucose-stimulated insulin secretion and improves peripheral insulin sensitivity^46^. Thus, it seems plausible that ghrelin changes after sleeve gastrectomy also contribute, at least in part, to the observed improvement in insulin sensitivity after sleeve gastrectomy.

Obesity is associated with an increased release of FFA from adipocytes due to increased lipolysis^7^,^47^. Circulating FFA are taken up by the liver and metabolized by two main pathways: i) the mitochondrial β-oxidation to generate ATP; and ii) esterification to produce TG, which can be either incorporated into very-low density lipoprotein (VLDL) particles or stored within the hepatocytes^48^. AMPK coordinates the changes in the activity of enzymes of hepatic lipid metabolism through the regulation of the partitioning of FFA both in oxidative and biosynthetic pathways with defects in this pathway leading to the development of NAFLD^49^,^50^. In the present study, lack of changes in the basal AMPK and ACC expression and activity as well as in the mitochondrial copy number in obese rats might reflect an impaired AMPK transduction signalling and dysfunction in mitochondrial biogenesis in the rat steatotic liver, which is in accordance with previous studies of our group^51^ and others^52^,^53^. Interestingly, a notable increase in hepatic AMPK and ACC phosphorylation was found after sleeve gastrectomy, which is in agreement with other studies showing similar changes in AMPK and ACC activation after bariatric surgery^54^. Accordingly, an upregulation of hepatic *Cpt1a* and its upstream molecule *Ppara* as well as an increase in mitochondrial content was observed after sleeve gastrectomy, suggesting an increased flux of FFA towards mitochondrial β-oxidation and higher mitochondrial copy number rather than inducing *de novo* TG synthesis. Interestingly, ghrelin triggers AMPK signalling in rat ventricular cardiomyocytes, myoblasts, hypothalamic neurons and hepatocytes^18^,^55^,^56^,^57^. Analogously, we found that acylated and desacyl ghrelin promoted AMPK and ACC activation in primary rat hepatocytes, suggesting that this hormone may be involved in the molecular mechanisms whereby sleeve gastrectomy improves hepatic mitochondrial function. In addition, acylated ghrelin also upregulates *Ppara* and *Cpt1a* expression, while the mitochondrial content was not affected. This observation leads to the notion that acylated ghrelin should be considered an important effector improving mitochondrial β-oxidation efficiency, whereas the beneficial effects on mitochondrial biogenesis after the surgery are not mediated by this hormone.

Autophagy plays an important role in the regulation of hepatic lipid metabolism. *In vitro* and *in vivo* studies in murine hepatocytes and hepatic tissue have demonstrated that autophagy mediates the breakdown of lipid stores and that an inhibition of autophagy increases TG storage in lipid droplets leading to the development of a fatty liver^8^,^58^. The inhibition of autophagy sensitizes hepatocytes to palmitic acid-induced apoptosis, suggesting a pro-survival function of autophagy against lipotoxicity^59^. In this sense, decreased levels of autophagy have been found in the liver of severely obese leptin-deficient *ob/ob* mice as well as in obese, diabetic OLETF rats, which might promote lipid accumulation and impaired hepatic function in these animal models^8^,^60^. However, our results showed no changes in the hepatic gene expression of the autophagy-related factors *Atg5* and *Atg7,* in the formation of autophagosomes evidenced by the LC3B-II/I conversion or in the inhibition of autophagy, expressed as p62 accumulation in obese rats. In this regard, hepatic autophagy is influenced by the degree of hepatic steatosis that might change the autophagosome formation during the ongoing NAFLD in adult obese rats. We herein report, for the first time, that sleeve gastrectomy is associated with an upregulation of the autophagy-related genes *Atg5* and *Atg7* and the LC3B-II/I ratio as well as with a decrease in the autophagy inhibition marker p62, suggesting that the induction of autophagy after this surgical procedure improves hepatic lipid clearance via lipophagy. Ghrelin isoforms are involved in the regulation of autophagy in several tissues, including the adipose tissue, small intestine, skeletal muscle and the heart^38^,^61^,^62^,^63^. Mao and colleagues reported that ghrelin induces autophagy in the liver via the activation of the AMPK signalling pathway^56^, but the specific role of each isoform of the hormone remains unclear. Interestingly, *Mboat4*-knockout mice lacking the gene encoding GOAT, which catalyzes the acylation of ghrelin, show a marked reduction of hepatic autophagy during fasting, suggesting an important role of acylated ghrelin in the regulation of autophagy in the liver during energy depletion^35^. In the present study, acylated ghrelin increased *Atg5, Atg7* and the LC3B-II/I ratio and reduced p62 accumulation, while desacyl ghrelin only decreased p62 expression in hepatocytes, suggesting that acylated ghrelin and, to a lesser extent, desacyl ghrelin stimulate hepatic autophagy. Taken together, our findings suggest that the increase in acylated ghrelin levels after sleeve gastrectomy is involved in the induction of liver autophagy.

In summary, we herein show that the beneficial effects of bariatric surgery on NAFLD are mediated via a decrease in lipogenesis as well as an increase in autophagy and mitochondrial β-oxidation. The decrease in desacyl ghrelin after sleeve gastrectomy contributes to the reduction of lipogenesis, whereas the increased acylated ghrelin levels activate AMPK-activated mitochondrial FFA β-oxidation and autophagy in obese rats. These results support the notion that both ghrelin isoforms constitute key elements involved in the improvement of NAFLD after bariatric surgery.

## Methods

### Experimental animals and study design

Four-week-old male Wistar rats (n = 161) were fed *ad libitum* during 4 months with either a normal diet (ND) (n = 22) or a high-fat diet (HFD) (n = 139). Obese rats were randomized into weight-matched groups to be submitted either to the sleeve gastrectomy (n = 37) or a sham operation (n = 41). Anesthesia, sham surgery and sleeve gastrectomy were performed as earlier described^64^. Following the surgical interventions, a group of animals were fed *ad libitum* a HFD, while another group was switched to a ND. In order to discriminate the effects of a reduced food intake following the bariatric surgery, two groups of obese rats were pair-fed to the amount of food eaten by the animals undergoing the sleeve gastrectomy switched to either the ND (n = 17) or maintaining the HFD (n = 23). Four weeks after the surgical and dietary interventions, rats were killed by decapitation after an 8-h fast. The methods were carried out in accordance with the relevant guidelines and regulations. All experimental procedures were approved by the Ethical Committee for Animal Experimentation of the University of Navarra (049/10) and conformed to the European Guidelines for the Care and Use of Laboratory Animals (directive 2010/63/EU).

### Blood and tissue analysis

Serum alanine aminotransferase (ALT), aspartate aminotransferase (AST), and intrahepatic TG were determined by enzymatic methods, as previously described^65^. Fasting acylated and desacyl ghrelin levels were also assessed using a rat/mouse EIA Kit (A05117 and A05118, Cayman Chemical, Ann Harbor, MI, USA).

### RNA isolation and real-time PCR

RNA isolation and purification were performed as earlier described^66^. Transcript levels of *Atg5, Atg7, Cav1, Cd36, Cpt1a, Dgat1, Fasn, Ghrl, Mboat4, Mogat2, Ppara, Pparg, Sqstm1, Srebf1* and *Tfam* (Supplementary Table S1) were quantified by real-time PCR (7300 Real Time PCR System, Applied Biosystems, Foster City, CA, USA). All results were normalized for the expression of *18* *S* rRNA (Applied Biosystems), and relative quantification was calculated as fold expression over the calibrator sample^66^.

### Western-blot studies

Blots were incubated overnight at 4 °C with rabbit polyclonal anti-ACC, rabbit polyclonal anti-phospho-ACC (Ser79), rabbit polyclonal anti-AMPKα, rabbit monoclonal anti-phospho-AMPKα (Thr172), rabbit monoclonal anti-CPT1A, rabbit polyclonal anti-FAS, rabbit polyclonal anti-LC3B (Cell Signaling Technology, Inc., Danvers, MA, USA), rabbit polyclonal anti-p62 (Sigma St. Louis, MO, USA) antibodies or murine monoclonal anti-β-actin (Sigma). The antigen-antibody complexes were visualized using horseradish peroxidase (HRP)-conjugated secondary antibodies and the ECL Plus detection system (Amersham Biosciences, Buckinghamshire, UK).

### Immunohistochemistry of adipophilin

The immunodetection of the lipid droplet-marker adipophilin in liver sections was performed by the indirect immunoperoxidase method^65^, using a mouse monoclonal anti-adipophilin (Acris, Hiddenhausen, Germany) antibody. A semi-quantitative evaluation of steatosis in adipophilin-stained liver histological sections was performed. Hepatic steatosis was scored according to the staining of adipophilin by three blinded expert observers as: (0) none; (1) mild; (2) moderate; or (3) severe steatosis.

### DNA extraction and analysis of mitochondrial DNA amount

The amount of mitochondrial DNA (mtDNA), extracted and purified using DNeasy Blood and Tissue Kit (Qiagen, Barcelona, Spain), was determined by real-time PCR of the mitochondrial cytochrome B (*Cytb*) gene normalized to the nuclear β-actin (*Actb*) gene (Supplementary Table S1), as previously described^67^. The real-time PCR was performed with 25 ng of total DNA using the TaqMan^®^ Universal PCR Master Mix (Applied Biosystems), according to the manufacturer’s instructions.

### Cell cultures

Primary rat hepatocytes were purchased from Tebu-Bio (Barcelona, Spain) and cultured in a collagen sandwich configuration^68^. Rat hepatocytes were seeded into 6-well plates (3 × 10^5^ cells/well) and grown in Complete Growth Medium [DMEM/F-12 medium (Invitrogen) supplemented with 10% fetal bovine serum (FBS), 5 μg/mL insulin, 5 μg/mL transferrin, 5 ng/mL selenium (Invitrogen, Paisley, UK), 40 ng/mL dexamethasone (Sigma), 20 ng/mL epidermal growth factor (Sigma) and antibiotic-antimycotic] for 24 h. Then, cells were serum-starved for 24 h and stimulated with increasing concentrations (10, 100, 1000 pmol/L) of acylated and desacyl ghrelin, (Tocris, Ellisville, MO, USA) for 24 h. These physiological and supraphysiological concentrations of ghrelin isoforms to carry out the experiments were chosen on the basis of previous studies performed in our laboratory^20^,^38^.

### Statistical analysis

Data are expressed as the mean ± SEM. Statistical differences between mean values were analyzed using Student’s *t* test, two-way ANOVA (diet × surgery), one-way ANOVA followed by Tukey’s or Dunnett’s *post-hoc* tests or the non-parametric Kruskal-Wallis test followed by *U* Mann-Whitney’s pairwise comparisons, where appropriate. Pearson’s correlation coefficients (*r*) were used to analyze the association between variables.

For more detailed Methods see Supplementary information.

## Additional Information

**How to cite this article**: Ezquerro, S. *et al*. Acylated and desacyl ghrelin are associated with hepatic lipogenesis, β-oxidation and autophagy: role in NAFLD amelioration after sleeve gastrectomy in obese rats. *Sci. Rep.* **6**, 39942; doi: 10.1038/srep39942 (2016).

**Publisher's note:** Springer Nature remains neutral with regard to jurisdictional claims in published maps and institutional affiliations.

## Supplementary Material

Supplementary Material

## Figures and Tables

**Table 1:** Body weight, insulin sensitivity and markers of hepatic function of lean and diet-induced obese rats.

Determination	ND (n = 22)	HFD (n = 21)	*P*
Body weight (g)	481 ± 15	606 ± 17	**<0.001**
Whole-body white adiposity (g)	33 ± 3	77 ± 6	**<0.001**
Glucose (mg/dL)	80 ± 2	90 ± 2	**<0.01**
Insulin (ng/mL)	2.1 ± 0.4	3.7 ± 0.6	**<0.05**
HOMA	0.52 ± 0.11	0.96 ± 0.17	**<0.05**
Liver (g)	12.3 ± 0.5	14.5 ± 0.5	**<0.05**
Intrahepatic TG (mg/g)	23.9 ± 1.6	34.7 ± 2.3	**<0.001**
AST (IU/L)	36 ± 3	41 ± 3	>0.05
ALT (IU/L)	10 ± 1	11 ± 1	>0.05

ND, normal diet; HFD, high-fat diet; TG, triacylglycerols. Data are the mean ± S.E.M. Statistical differences were analyzed by Student’s *t* test.

**Table 2:** Body weight, insulin sensitivity and markers of hepatic function four weeks after surgical and dietary interventions in diet-induced obese rats.

Determination	Sham ND (n = 17)	Sleeve ND (n = 15)	Pair-fed ND (n = 17)	Sham HFD (n = 24)	Sleeve HFD (n = 22)	Pair-fed HFD (n = 23)
Body weight (g)	503 ± 10	478 ± 11^b^	495 ± 10	549 ± 18^a^	514 ± 13^a,b^	554 ± 13^a^
Total weight loss (%)	14 ± 1	18 ± 1^b^	12 ± 1	3 ± 1^a^	10 ± 2^a,b^	0 ± 1^a^
Whole-body white adiposity (g)	41 ± 3	37 ± 3^b^	49 ± 3	58 ± 7^a^	55 ± 1^a,b^	64 ± 3^a^
Glucose (mg/dL)	79 ± 2	78 ± 2^b^	90 ± 3	101 ± 4^a^	103 ± 3^a,b^	111 ± 5^a^
Insulin (ng/mL)	2.4 ± 0.4	1.6 ± 0.3^b^	2.2 ± 0.3	2.2 ± 0.3	1.8 ± 0.2^b^	3.1 ± 0.4
HOMA	0.52 ± 0.10	0.44 ± 0.07^b^	0.56 ± 0.08	0.66 ± 0.01^a^	0.55 ± 0.05^a,b^	1.00 ± 0.16^a^
Liver (g)	12.4 ± 0.5	11.0 ± 0.5^b^	12.1 ± 0.4	13.4 ± 0.5^a^	12.1 ± 0.4^a,b^	12.4 ± 0.4^a^
Intrahepatic TG (mg/g)	23.4 ± 1.3	21.9 ± 1.6^b^	23.2 ± 1.6	35.0 ± 0.4^a^	25.3 ± 1.7^a,b^	31.3 ± 2.3^a^
AST (IU/L)	41 ± 3	26 ± 2^b^	37 ± 2	40 ± 3	32 ± 2^b^	36 ± 3
ALT (IU/L)	12 ± 1	9 ± 1	11 ± 2	11 ± 1	10 ± 1	9 ± 2

ND, normal diet; HFD, high-fat diet; TG, triacylglycerols. Data are the mean ± S.E.M. Statistical differences were analyzed by two-way ANOVA. ^a^*P* < 0.05 effect of diet; ^b^*P* < 0.05 effect of surgery.
