# Supplementary material for: Acylated and desacyl ghrelin are associated with hepatic lipogenesis, β-oxidation and autophagy: role in NAFLD amelioration after sleeve gastrectomy in obese rats
Source: Sci Rep. 2016 Dec 23;6:39942. doi: 10.1038/srep39942 (PMC5180230; doi:10.1038/srep39942)
Supplement: Supplementary Material [file srep39942-s1.doc]

Acylated and desacyl ghrelin are associated with hepatic lipogenesis, β-oxidation and autophagy: role in NAFLD amelioration after sleeve gastrectomy in obese rats

Silvia Ezquerro^1,5,6^, Leire Méndez-Giménez^1,5,6^, Sara Becerril^1,5,6^, Rafael Moncada^2,5,6^, Víctor Valentí^3,5,6^, Victoria Catalán^1,5,6^, Javier Gómez-Ambrosi^1,5,6^, Gema Frühbeck^1,4,5,6^, Amaia Rodríguez^1,5,6,*^

^1^Metabolic Research Laboratory, ^2^Department of Anesthesia, ^3^Department of Surgery, and ^4^Department of Endocrinology & Nutrition, Clínica Universidad de Navarra, Pamplona, Spain; ^5^CIBER Fisiopatología de la Obesidad y Nutrición (CIBEROBN), Instituto de Salud Carlos III, Madrid, Spain; ^6^Obesity & Adipobiology Group, Instituto de Investigación Sanitario de Navarra (IdiSNA), Pamplona, Spain.

**Word count:** Abstract (200); Main text (3,355); Table (2); Figures (5); Supplemental data (2); References (69).

***Corresponding author:**

Amaia Rodríguez, PhD

Metabolic Research Laboratory

Clínica Universidad de Navarra

Irunlarrea 1

31008 Pamplona (Spain)

Phone: +34 948 42 56 00 (ext. 3357)

e-mail: [arodmur@unav.es](mailto:arodmur@unav.es)

**Supplementary methods**

**Experimental animals and study design.** Four-week-old male Wistar rats (n=161) (breeding house of the University of Navarra) were housed in individual cages in a room with controlled temperature (22±2 ºC), ventilation (at least 15 complete air changes/h), 12:12-h light-dark cycle (lights on at 8:00 am) and relative humidity (50± 10%) under pathogen-free conditions. Animals were fed *ad libitum* during 4 months with either a normal diet (ND) (n=22) (12.1 kJ: 4% fat, 82% carbohydrate and 14% protein, diet 2014S, Harlan, Teklad Global Diets, Harlan Laboratories Inc., Barcelona, Spain) or a high-fat diet (HFD) (n=139) (23.0 kJ/g: 59% fat, 27% carbohydrate and 14% protein, diet F3282; Bio-Serv, Frenchtown, NJ, USA). Body weight and food intake were registered weekly to monitor the progression of DIO rats that reached a mean body weight of 630±15 g after 4 months on the HFD.

Obese rats were randomized into weight-matched groups to be submitted either to the sleeve gastrectomy (n=37) or a sham operation (n=41). Anesthesia and sleeve gastrectomy were performed according to previously described methodology^1-3^. Briefly, for the sleeve gastrectomy a laparotomy incision was made in the abdominal wall and the stomach was isolated outside the abdominal cavity. Loose gastric connections to the spleen and liver were released along the greater curvature and the great omentum was ligated and divided down to the level of the pylorus. About 60-70% of the forestomach and glandular stomach was excised out using an automatic stapler (AutoSuture TA DST Series, Tyco Healthcare group LP, Norwalk, CT, USA) with a TA30V3L load, leaving a tubular gastric remnant in continuity with the esophagus upwards and the pylorus and duodenum downwards. The sham surgery comprised the same laparotomic incision as well as handling of the stomach except for the gastrectomy. Following the surgical interventions [sham operation (n=24) or sleeve gastrectomy (n=22)], a group of obese animals continued to be fed *ad libitum* a HFD, while another group of obese rats was switched to a ND *ad libitum* [sham operation (n=17) or sleeve gastrectomy (n=15)]. In order to discriminate the effects of a reduced food intake following the bariatric surgery, two groups of obese rats were pair-fed to the amount of food eaten by the animals undergoing the sleeve gastrectomy switched to either the ND or the HFD [pair-fed ND (n=17) or pair-fed HFD (n=23)]. Four weeks after the surgical and dietary interventions, rats were killed by decapitation after an 8-h fasting period. The liver, epididymal (EWAT), perirenal (PRWAT) and subcutaneous (SCWAT) white adipose tissues were carefully dissected out, weighed, frozen in liquid nitrogen and stored at -80 ºC until processed for the study. A small portion of the tissue was fixed in 4% paraformaldehyde for histological analyses. Blood samples were immediately collected and sera were obtained by cold centrifugation (4 ºC) at 700 *g* for 15 min. The percentage of total weight loss (%TWL) was calculated from the equation %TWL= (pre-interventional body weight- post-interventional body weight)/pre-interventional body weight x 100. All experimental procedures conformed to the European Guidelines for the care and use of Laboratory Animals (directive 2010/63/EU) and were approved by the Ethical Committee for Animal Experimentation of the University of Navarra (049/10).

**Blood and tissue analysis.** Serum glucose was determined by an automatic glucose sensor (Ascencia Elite, Bayer, Barcelona, Spain). Insulin was determined by ELISA (Crystal Chem, Inc., Chicago, IL, USA). Intra- and inter-assay coefficients of variation for measurements of insulin were 3.5%, for the former, and 5.4%, for the latter. Insulin resistance was calculated using the homeostasis model assessment (HOMA), calculated with the formula: fasting insulin (μU/mL) × fasting glucose (mmol/L)/22.5). Serum alanine aminotransferase (ALT), aspartate aminotransferase (AST), FFA and TG were measured by enzymatic methods using commercially available kits (Infinity^TM^, Thermo Electron Corporation, Melbourne, Australia). Intrahepatic TG was determined by enzymatic methods, as previously described^4^. Fasting acylated and desacyl ghrelin levels were also assessed using a rat/mouse EIA Kit (A05117 and A05118**,** Cayman Chemical, Ann Harbor, MI, USA). Intra- and inter-assay coefficients of variation for measurements were 5.7% and 6.1%, respectively, for the former and 5.2% and 5.5 %, for the latter.

**RNA isolation and real-time PCR.** RNA isolation and purification were performed as earlier described^5^. Transcript levels of *Atg5, Atg7,* *Cav1, Cd36, Cpt1a, Dgat1, Fasn*, *Ghrl, Mboat4,* *Mogat2, Ppara, Pparg, Sqstm1,* *Srebf1* and *Tfam* were quantified by real-time PCR (7300 Real Time PCR System, Applied Biosystems, Foster City, CA, USA). Primers and probes (**Supplementary Table 1**) were designed using the software Primer Express 2.0 (Applied Biosystems) and acquired from Genosys (Sigma. St. Louis, MO, USA). Primers or TaqMan® probes encompassing fragments of the areas from the extremes of two exons were designed to ensure the detection of the corresponding transcript avoiding genomic DNA amplification. The cDNA was amplified at the following conditions: 95 ºC for 10 min, followed by 45 cycles of 15 s at 95 ºC and 1 min at 59 ºC, using the TaqMan® Universal PCR Master Mix (Applied Biosystems). The primer and probe concentrations were 300 and 200 nmol/L, respectively. All results were normalized for the expression of *18S* rRNA (Applied Biosystems), and relative quantification was calculated as fold expression over the calibrator sample^5^. All samples were run in triplicate and the average values were calculated.

**Western-blot studies.** Tissues were harvested and homogenized in ice-cold lysis buffer (0.1% SDS, 1% Triton X-100, 5 mmol/L EDTA·2H_2_O, 1 mol/L Tris-HCL, 150 mmol/L NaCl, 1% sodium deoxycholate, pH 7.40) complemented with a protease inhibitor cocktail (Complete^TM^ Mini-EDTA free, Roche, Mannheim, Germany). Lysates were centrifuged at 12,000 *g* at 4 ºC for 15 min to remove nuclei and unruptured cells. Total protein concentrations were determined by the Bradford assay^6^, using bovine serum albumin (BSA) (Sigma) as standard. Thirty micrograms of total protein of liver homogenates were diluted in loading buffer 4X (20% β-mercaptoethanol, 40 mmol/L dithiothreitol, 8% SDS, 40% glycerol, 0.016% bromophenol blue, 200 mmol/L Tris-HCl, pH 6.80) and heated for 10 min at 100 ºC. Samples were run out in Mini-PROTEAN® TGX^TM^ precast gels (Bio-Rad Laboratories, Inc., Hercules, CA, USA), subsequently transferred to nitrocellulose membranes (Bio-Rad) and blocked in Tris-buffered saline (10 mmol/L Tris-HCl, 150 mmol/L NaCl, pH 8.00) with 0.05% Tween 20 (TBS-T) containing 5% non-fat dry milk for 1 h at room temperature (RT). Membranes were then incubated overnight at 4 ºC with rabbit polyclonal anti-ACC, rabbit polyclonal anti-phospho-ACC (Ser79), rabbit polyclonal anti-AMPKα, rabbit monoclonal anti-phospho-AMPKα (Thr172), rabbit monoclonal anti-CPT1A, rabbit polyclonal anti-FAS, rabbit polyclonal anti-LC3B (Cell Signaling Technology, Inc., Danvers, MA, USA), rabbit polyclonal anti-p62 (Sigma) antibodies (diluted 1:5,000 for ACC, phospho-ACC, FAS, LC3B and p62 and 1:2,000 for AMPK, phospho-AMPK and CPT1A in blocking solution) or murine monoclonal anti-β-actin (Sigma) (diluted 1:5,000 in blocking solution). The antigen-antibody complexes were visualized using horseradish peroxidase (HRP)-conjugated anti-rabbit or anti-mouse IgG antibodies (diluted 1:5,000 for ACC, phospho-ACC, FAS, LC3B, p62 and β-actin and 1:2,000 for AMPK, phospho-AMPK and CPT1A in blocking solution) and the enhanced chemiluminescence ECL Plus detection system (Amersham Biosciences, Buckinghamshire, UK). The intensity of the bands was determined by densitometric analysis with the Gel Doc^TM^ gel documentation system and the Quantity One 4.5.0 software (Bio-Rad) and normalized with β-actin density values.

**Immunohistochemistry of adipophilin.** The immunodetection of the specific marker of lipid accumulation adipophilin in histological sections of liver was performed by the indirect immunoperoxidase method, as previously described^4,7^. Sections of formalin-fixed paraffin-embedded liver (4 μm) were dewaxed in xylene, rehydrated in decreasing concentrations of ethanol and treated with 3% H_2_O_2_ (Sigma) in absolute methanol for 10 min at RT to quench endogenous peroxidase activity. Slides were blocked during 60 min with 1% goat serum (Sigma) diluted in Tris-buffer saline (TBS) (50 mmol/L Tris, 0.5 mol/L NaCl, pH 7.36) to prevent non-specific absorption. Sections were incubated overnight at 4 ºC with mouse monoclonal anti-adipophilin (Acris, Hiddenhausen, Germany) antibody diluted 1:200 in TBS. After washing three times with TBS (5 min each), slides were incubated with DAKO Real^TM^ EnVision^TM^ anti-rabbit/mouse (K5007; Dako, Golstrup, Denmark) for 1 h at RT. The peroxidase reaction was visualized using a 0.5 mg/mL diaminobenzidine (DAB)/0.03% H_2_O_2_ solution diluted in 50 mmol/L Tris-HCl, pH 7.36, and Harris hematoxylin solution (Sigma) as counterstaining. Negative control slides without primary antibody were included to assess non-specific staining. A semi-quantitative evaluation of steatosis in adipophilin-stained liver histological sections was performed. Hepatic steatosis was scored according to the staining of adipophilin by three blinded expert observers as: (0) none; (1) mild; (2) moderate; or (3) severe steatosis.

**DNA extraction and analysis of mtDNA amount**. The amount of mtDNA, extracted and purified using DNeasy Blood and Tissue Kit (Qiagen, Barcelona, Spain), was determined by real-time PCR of the mitochondrial cytochrome B (CYTB) gene normalized to the nuclear β-actin (ACTB) gene^8^. The RT-PCR was performed with 25 ng of total DNA using the TaqMan® Universal PCR Master Mix (Applied Biosystems), according to the manufacturer’s instructions. The RT-PCR data were analyzed by the comparative “ΔΔCt method”. The primer sequences are available in **Supplementary Table S1**.

**Cell cultures.** Non tumorigenic rat hepatocytes were purchased from Tebu-Bio (Barcelona, Spain). Cells were maintained in DMEMF-12 medium (Invitrogen, Paisley, UK) supplemented with 10% fetal bovine serum (FBS), 5 μg/mL insulin, 5 μg/mL transferrin, 5 ng/mL selenium (Invitrogen), 40 ng/mL dexamethasone (Sigma), 20 ng/mL epidermal growth factor (Sigma) and antibiotic-antimycotic (Complete Growth Medium). Rat hepatocytes were cultured in a collagen sandwich configuration as described by Shulman *et al*^9^, were seeded into 6-well plates at 3x10^5^ cells/well and grown in Complete Growth Medium for 24h. Cells were serum starved for 24 h and then treated for 24 h with increasing concentrations (10, 100, 1000 pmol/L) of acylated ghrelin or desacyl ghrelin (Tocris, Ellisville, MO, USA) for 24 h. These physiological and supraphysiological concentrations of ghrelin isoforms to carry out the experiments were chosen on the basis of previous studies performed in our laboratory^10,11^.

**Statistical analysis.** Data are expressed as the mean ± SEM. Statistical differences between mean values were analyzed using Student’s *t* test, two-way ANOVA (diet x surgery), one-way ANOVA followed by Tukey’s or Dunnett’s *post-hoc* tests or the non-parametric Kruskal-Wallis test followed by *U* Mann-Whitney´s pairwise comparisons, where appropriate. Pearson’s correlation coefficients (*r*) were used to analyze the association between variables. The statistical analyses were performed using the SPSS/Windows version 15.0 software (SPSS Inc., Chicago, IL, USA).

**References**

1. Valentí, V. *et al.* Sleeve gastrectomy induces weight loss in diet-induced obese rats even if high-fat feeding is continued. *Obes Surg*. **21**, 1438-1443 (2011).

2. Rodríguez, A. *et al.* Sleeve gastrectomy reduces blood pressure in obese (*fa/fa*) Zucker rats. *Obes Surg*. **22**, 309-315 (2012).

3. Rodríguez, A. *et al.* Short-term effects of sleeve gastrectomy and caloric restriction on blood pressure in diet-induced obese rats. *Obes Surg*. **22**, 1481-1490 (2012).

4. Rodríguez, A. *et al.* Reduced hepatic aquaporin-9 and glycerol permeability are related to insulin resistance in non-alcoholic fatty liver disease. *Int J Obes*. **38**, 1213-1220 (2014).

5. Catalán, V. *et al.* Validation of endogenous control genes in human adipose tissue: relevance to obesity and obesity-associated type 2 diabetes mellitus. *Horm Metab Res*. **39**, 495-500 (2007).

6. Bradford, M. M. A rapid and sensitive method for the quantitation of microgram quantities of protein utilizing the principle of protein-dye binding. *Anal Biochem*. **72**, 248-254 (1976).

7. Rodríguez, A. *et al.* Insulin- and leptin-mediated control of aquaglyceroporins in human adipocytes and hepatocytes is mediated via the PI3K/Akt/mTOR signaling cascade. *J Clin Endocrinol Metab*. **96**, E586-597 (2011).

8. Heinonen, S. *et al.* Impaired mitochondrial biogenesis in adipose tissue in acquired obesity. *Diabetes*. **64**, 3135-3145 (2015).

9. Shulman, M. & Nahmias, Y. Long-term culture and coculture of primary rat and human hepatocytes. *Methods Mol Biol*. **945**, 287-302 (2013).

10. Rodríguez, A. *et al.* Acylated and desacyl ghrelin stimulate lipid accumulation in human visceral adipocytes. *Int J Obes*. **33**, 541-552 (2009).

11. Rodríguez, A. *et al.* The ghrelin *O*-acyltransferase-ghrelin system reduces TNF-alpha-induced apoptosis and autophagy in human visceral adipocytes. *Diabetologia*. **55**, 3038-3050 (2012).

**Supplementary Table S1. Sequences of primers and TaqMan®** probes.

| Gene  (GenBank accession no.) | Oligonucleotide sequence (5’-3’) | | Nucleotides | |  |
| --- | --- | --- | --- | --- | --- |
| Actb  (NM_005111) |  | |  | |  |
| Forward | GGGAAATCGTGCGTGACATT | | 2188-2207 | |  |
| Reverse | GCTATGAGCTGCCTGACGGT | | 2287-2306 | |  |
| Taqman® probe | FAM-TGCCCTAGACTTCGAGCAAGAGATGGC-TAMRA | | 2228-2254 | |  |
| Atg5  (NM_001014250.1) |  | |  | |  |
| Forward | CACACCCCTGAAATGGCATTAT | | 449-470 | |  |
| Reverse | ttccagaaaaggaccttctgca | | 547-568 | |  |
| Taqman® probe | FAM- TGCATCAAGCTCAGCT-TAMRA | | 497-512 | |  |
| Atg7  (NM_001012097.1) |  | |  | |  |
| Forward | GGTCCGAGGATGGTGAACCT | | 940-959 | |  |
| Reverse | CTGAATCTCAAGCTGATGTGCT | | 1006-1027 | |  |
| Taqman® probe | FAM-ATGGACCCCAAAAGGC-TAMRA | | 970-985 | |  |
| Cav1  (NM_031556.3) |  | |  | |  |
| Forward | ACGACGACGTGGTCAAGATTG | | 334-354 | |  |
| Reverse | ttggttttaccgcttgctgtct | | 446-467 | |  |
| Taqman® probe | FAM-TGCGGAACCAGAAGGGACACACAGTTT-TAMRA | | 371-397 | |  |
| Cd36  (NM_031561.2) |  | |  | |  |
| Forward | GACATTTGCAGGTCCATCTATGC | | 1041-1064 | |  |
| Reverse | cagaacccagacaaccactgttt | | 1146-1168 | |  |
| Taqman® probe | FAM-TTCTTCCAGCCAACGCCTTTGCCT-TAMRA | | 1111-1137 | |  |
| Cpt1a  (NM_031559) |  | |  | |  |
| Forward | AACTTTGTGCAGGCCATGATG | | 1942-1961 | |  |
| Reverse | AGCTTGTGAGAAGCACCAGCA | | 2007-2026 | |  |
| Taqman® probe | FAM-ACCCCAAGTCAACGGCAGAGCAGA-TAMRA | | 1964-1977 | |  |
| Cytb  (NC_001665.2) |  | |  | |  |
| Forward | GCCTTCCTACCATTCCTGCATA | | 904-925 | |  |
| Reverse | TTAACATGAATCGGAGGCCAA | | 1001-1023 | |  |
| Taqman® probe | FAM-CAAAACAACGCAGCTTAACATTCCGCC-TAMRA | | 929-955 | |  |
| Dgat1  (NM_053437.1) |  | |  | |  |
| Forward | CGGTCCCCAACCATCTGATAT | | 1048-1068 | |  |
| Reverse | tttccactcatgtctcaatgctgtggca | | 1132-1152 | |  |
| Taqman® probe | FAM-TTCCACTCATGTCTCAATGCTGTGGCA-TAMRA | | 1091-2018 | |  |
| Fasn  (NM_017332.1) | |  | |  | |
| Forward | | AGAGCATTCTGGCCACATCCT | | 4349-4369 | |
| Reverse | | tgtctccgaaaagagccgg | | 4435-4453 | |
| Taqman® probe | | FAM-CCCAGCCTGTGTGGCTAACAGCCAT-TAMRA | | 4373-4397 | |
| Ghrl  (NM_021669.2) | |  | |  | |
| Forward | | AAGCCCAGCAGAGAAAGGAATC | | 130-151 | |
| Reverse | | tcaatgctcccttcgatgttg | | 256-276 | |
| Taqman® probe | | FAM-AACTGCAGCCACGAGCTCTGGAAGG-TAMRA | | 169-193 | |
| Ghsr  (NM_032075.3) | |  | |  | |
| Forward | | CAGAACCACAAGCAGACAGTGAA | | 760-782 | |
| Reverse | | agatacctcttttccaagtccttcg | | 841-865 | |
| Taqman® probe | | FAM-ATGCTTGCTGTGGTGGTGTTT-TAMRA | | 784-804 | |
| Mboat4  (NM_001107317.2) | |  | |  | |
| Forward | | CACCTGGGTCTTCACTACAAGGA | | 292-314 | |
| Reverse | | acatttctgaagggaaggtggag | | 407-429 | |
| Taqman® probe | | FAM-tccccctgtgaggttctacatcac-TAMRA | | 333-356 | |
| Mogat2  (NM_001109436.2) | |  | |  | |
| Forward | | TCCCTGTCTCTTTGGTCAAGACA | | 289-311 | |
| Reverse | | ttcttaacctgtgcactgaaagca | | 382-405 | |
| Taqman® probe | | FAM-CGGAACTACATCGCAGGCTTTCACCC-TAMRA | | 330-355 | |
| Ppara  (NM_013196.1) | |  | |  | |
| Forward | | AAGGCCTCAGGATACCACTATGG | | 699-721 | |
| Reverse | | CAGCTTCGATCACACTTGTCGTA | | 783-805 | |
| Taqman® probe | | FAM-CTGCAAGGGCTTCTTTCGGCGAAC-TAMRA | | 740-763 | |
| Pparg  (NM_013124) | |  | |  | |
| Forward | | CTGACCCAATGGTTGCTGATTAC | | 257-279 | |
| Reverse | | CCTGTTGTAGAGTTGGGTTTTTTCA | | 351-375 | |
| Taqman® probe | | FAM-TGAAGCTCCAAGAATACCAAAGTGCG-TAMRA | | 290-315 | |
| Sqstm1  (NM_175843) | |  | |  | |
| Forward | | GCTCTCTAGACCCCTCACAGGAA | | 1125-1147 | |
| Reverse | | CAGATGCTGTCCATGGGTTTCT | | 1229-1250 | |
| Taqman® probe | | FAM-ACAGGGCTGAAGGAAGCTGCCCTGT-TAMRA | | 1154-1178 | |
| Srebf1  (NM_001276707.1) | |  | |  | |
| Forward | | ATGCGGCTGTCGTCTACCAT | | 2050-2069 | |
| Reverse | | AGTGTGCAGGAGATGCTATATCCAT | | 2158-2182 | |
| Taqman® probe | | FAM-CATGCCATGGGCAAGTACACAGGAGG-TAMRA | | 2085-2110 | |
| Tfam  (NM_031326.1) | |  | |  | |
| Forward | | ACACCCAGATGCAAAAGTTTCAG | | 315-337 | |
| Reverse | | AAGCTGAGTGGAAGGTGTACAAAGA | | 413-437 | |
| Taqman® probe | | FAM-AAAAATTGCAGCCATGTGGAGGGAGC-TAMRA | | 348-373 | |

*Actb*, β-actin; *Atg*, autophagy related; *Cav1*, caveolin 1; *Cd36,* CD36 molecule (thrombospondin receptor); *Cpt1a*, carnitine palmitoyl transferase 1a (liver); *Cytb,* cytochrome b; *Dgat1,* diacylglycerol *O*-acyltransferase 1; *Fasn,* fatty acid synthase; *Ghrl,* ghrelin; *Ghsr,* growth hormone secretagogue receptor; *Mboat4,* membrane bound *O*-acyltransferase domain containing 4; *Mogat2,* monoacylglycerol *O*-acyltransferase 2; *Ppara*, peroxisome proliferator-activator receptor α; *Pparg,* peroxisome proliferator-activator receptor γ; *Sqstm1,* sequestosome 1; *Srebf1*, sterol regulatory element binding factor 1c; *Tfam,* transcription factor A mitochondrial.

**Supplemental Fig. 1. Semi-quantitative evaluation of hepatic steatosis according to adipophilin staining.** Impact of obesity (A) and sleeve gastrectomy (B) on liver steatosis assessed by the staining of adipophilin in liver histological sections in a blind study. Differences were analyzed by χ^2^ test. **P*<0.05 *vs* lean control ND or sham-operated ND.
